# Long-term examination of pain and health-related outcomes in people with fibromyalgia before, during, and after COVID-19

**DOI:** 10.1097/PR9.0000000000001380

**Published:** 2025-12-18

**Authors:** Benjamin Mosch, Martin Diers

**Affiliations:** Clinical and Experimental Behavioral Medicine, Department of Psychosomatic Medicine and Psychotherapy, LWL University Hospital, Ruhr University Bochum, Bochum, Germany

**Keywords:** COVID-19, Pandemic, Psychosocial factors, Fibromyalgia, Chronic pain, Longitudinal

## Abstract

**Introduction:**

Since the outbreak of COVID-19, many studies have investigated long-term consequences associated to the pandemic. Undoubtedly, restrictions and changes in everyday life during that time have had a broad impact on mental health and well-being, particularly in vulnerable populations, such as chronic pain patients.

**Objectives:**

The aim of this study was to examine longitudinal progression of pain-related outcomes in patients with fibromyalgia (FM).

**Methods:**

Using a repeated measures design with comparative data acquired before (T1) and during the pandemic (T2), as well as data from a recent survey (T3), this study investigated long-term development of pain symptoms and well-being in FM (N = 75) throughout and beyond the pandemic. For this purpose, longitudinal changes of clinical parameters, such as pain severity, disability, and depressive mood, as well as self-perceived changes of pain, anxiety, depression, and physical activity were assessed.

**Results:**

Patients displayed significant self-perceived worsening of pain compared with both preceding survey periods. However, these self-perceived changes were not reflected in longitudinal increases of pain values. Pain-related interference, perceived helplessness, and social activity levels were significant predictors of pain severity at T3, when using only T3 determinants as predictors. On the other hand, with regard to the prepandemic and pandemic period, past pain levels (T1, T2) and affective distress experienced at T2 were significant predictors of T3 pain.

**Conclusions:**

These findings emphasize the importance of psychosocial factors for the long-term progression of pain symptoms. In addition, our data suggest remarkable long-term stability of pain symptoms, despite repeated evidence for a self-perceived worsening of pain.

## Abstract 2

Supplemental Digital Content is Available in the Text.

Patients with fibromyalgia report self-perceived worsening of pain but no longitudinal change of pain scores. Pain severity is predicted through multiple psychosocial factors.

## 1. Introduction

The COVID-19 (SARS-CoV-2) pandemic leads to several waves of public restrictions and widespread health-related concerns that have shaped social and personal life worldwide for around 2 years. Many studies reported negative trends in mental health and well-being, mainly increasing depressive symptoms, anxiety, and affective distress.^1,6,9,11,34^ This was particularly true for vulnerable groups such as people with chronic pain, who experienced more severe adverse effects.^4,7,12,23,25,33^ Chronic pain with comorbid depression and anxiety is most common among older individuals, also at a heightened risk of severe COVID-19 infections. In addition, Zanetti et al.^38^ observed increased mortality in rheumatic diseases. Given the restrictions that altered the lives of healthy individuals and patients with chronic pain, many studies are now asking: How can we assess the aftermath of the pandemic?

This current investigation ties in with 2 postal questionnaire surveys in 2019^2^ and 2022^25^ (T1 and T2). The 2022 study explored how patients with chronic pain were affected by restrictions and psychosocial burdens. A key distinction, which is also of relevance to this study, lies in the 2 types of symptom-related measures collected. First, subjectively perceived changes in symptoms were assessed. Second, current symptom severity was evaluated at each time point using standardized questionnaires, allowing these trajectories to be compared with self-perceived developments. The results indicated a significant self-perceived worsening of pain, depressive mood, anxiety, and reduced physical activity, assessed in a retrospective survey. Interestingly, these self-perceived changes were not reflected in longitudinal increases of pain-related test values or decreases of psychological well-being, assessed in a number of questionnaires at both time points (T1–T2). Pain severity at T1 best predicted severity at T2, while COVID-related outcomes showed no critical importance; only COVID-related fear predicted T2 pain. General perceived pandemic impact predicted self-perceived worsening. Patients with less severe prepandemic pain displayed greater longitudinal worsening.

This marks the starting point for our current examination. First, longitudinal development and retrospective evaluation were examined separately. We then investigate continuing development of parameters in our fibromyalgia (FM) sample to see whether self-perceived adverse effects^12,25^ prove to be reversible and whether initial prepandemic levels reappear. We also assessed items adapted from our previous study assessing experiences related to the COVID-19 pandemic, and new items, related to the changed situation including geopolitical conflicts, such as the war in Ukraine. To better understand differences in patients' symptom severity at T3 and self-perceived worsening of pain (T2–T3) in relation with current and past risk factors, 3 stepwise regression analyses were conducted. The predictors included the additional behavioral and clinical instruments as well as several single items described in detail in the Methods section.

Compared with our T2 data, we expected patients with FM to report self-perceived decreases of pain severity, depressive symptoms, and anxiety and increases in physical activity. However, such trends might depend on affective distress caused by current conflicts. Since long-term pandemic adverse effects are discussed, we assumed improvements from T2 to T3 in self-perceived measures might be less pronounced than the worsening of from T1 to T2. We expected no longitudinal change of pain severity ratings.

## 2. Materials and methods

### 2.1. Participants and procedure

A total of 75 female patients with FM (aged 58.2 ± 7.63 years, range 29–72 years), mainly recruited through social media support groups in 2019, completed a postal paper survey. Participation was voluntary and not compensated. FM diagnoses were obtained by medical professionals and met Wolfe et al.^37^ criteria (Table 1).

**Table 1:** Longitudinal demographic, psychometric, and clinical characteristics of fibromyalgia patients (N = 75).

	T1		T2		T3		GLM results
	*M*	SD	*M*	SD	*M*	SD	*F* (*df*); *P*
Age (years)	53.01	7.67	55.93	7.52	58.2	7.63	
Pain duration, years	18.21	14.22	20.21	15.12	22.34	13.71	
CES-D	22.82	7.25	23.29	6.5	22.99	7.05	0.24 (2); 0.79
FSQ							
Symptom severity score	9.46	2.14	9.32	2.37	9.08	2.45	1.34 (2); 0.27
Widespread pain index	11.33	4.62	10.88	4.61	10.55	4.73	1.21 (1.8); 0.3
PRSS							
Catastrophizing	2.27	1.1	2.12	1.21	2.09	1.21	1.48 (2); 0.23
Coping	3.02	0.91	3.07	0.82	3.09	0.95	0.23 (2); 0.79
CPG							
Pain intensity	71.11	12.5	70.51	14.88	70.53	14.23	
Disability score	64.62	19.92	63.72	20.34	60.09	22.72	
Chronic pain grade	3.27	0.83	3.16	1.01	3.08	0.96	
MPI							
Pain severity	3.93	1.11	4.02	1.09	3.84	1.16	1.54 (2); 0.22
Interference	4.16	1.22	4.1	1.18	3.76	1.3	**6.28 (1.85); 0.003** *
Life control	3.21	1.36	3.2	1.36	3.33	1.24	0.42 (2); 0.66
Affective distress	3.61	1.36	3.43	1.37	3.22	1.2	**3.3 (2); 0.04** *
Social support	3.27	1.69	3.22	1.67	3.11	1.73	0.35 (2); 0.71
Punishing responses	1.32	1.6	1.44	1.7	1.25	1.25	0.35 (2); 0.71
Solicitous responses	3.03	1.67	3.28	1.66	3.3	1.63	1.54 (1.83); 0.22
Distracting responses	2.6	1.45	2.76	1.65	2.65	1.69	0.38 (1.83); 0.66
Social activities	2.38	0.94	2.12	0.92	2.18	0.96	**4.12 (1.8); 0.022** *
General activity level	7.61	2.4	7.09	2.39	7.15	2.34	**3.67 (2); 0.03** *
PCS							
Helplessness			10.51	6.18	9.32	6.34	
Magnification			4.05	2.97	3.84	2.85	
Rumination			6.8	4.53	5.78	4.56	
Total PCS			21.36	12.38	18.81	12.88	

All values reported above refer to the current T3 sample (N *=* 75).

* Significant change (*P* < 0.05), marked in bold.

CES-D, Center for Epidemiologic Studies Depression Scale; CPG, Chronic Pain Grade Scale; FSQ, Fibromyalgia Survey Questionnaire; GLM, general linear model; M, mean; MPI, West Haven-Yale Multidimensional Pain Inventory; PCS, Pain Catastrophizing Scale; PRSS, Pain-Related Self Statements Scale; SD, standard deviation; T1, Initial examination in 2019/2020; T2, Second examination in 2022; T3, Recent investigation in 2024/2025.

Comparative data came from 2 earlier studies (T1: 2019/2020 and T2: 2022). The included participants of the T1 survey were assessed between May 2019 and February 2020. The second survey (T2, March–July 2022) included COVID-19–related items. The current survey (T3, October 2024–January 2025) followed the same postal procedure. Of the initial 208 patients with FM at T1, we included 109 patients at T2. Of these 109 patients, for T3, 16 patients were unavailable, 4 declined, and 14 did not return questionnaires, leaving 75 participants at T3, each provided 3 complete data sets.

The study was approved by the ethics review board of the Medical Faculty, Ruhr University Bochum. All participants gave written informed consent.

### 2.2. Diagnostic and clinical assessment

All of the questionnaires used are valid and widely approved instruments that are suitable for longitudinal surveys. At all 3 time points, patients completed 2 universal multidimensional tools for pain assessment: The West Haven-Yale Multidimensional Pain Inventory (MPI)^22^ (German version: Flor et al.^15^) and the Chronic Pain Grade Scale (CPG),^35^ as well as a diagnostic tool developed specifically for FM: the Fibromyalgia Survey Questionnaire (FSQ).^18^ Situation-specific aspects of pain coping were measured using the Pain-Related Self Statements Scale (PRSS) (German version: FSS, Flor et al.^14^). In addition, depressive symptoms were assessed using the Center for Epidemiologic Studies Depression Scale (CES-D^30^; German version: ADS) (Table 1). At T2 and T3, we additionally assessed the pain-catastrophizing scale (PCS).^24^ An overview of all single items capturing the individual perception of topics such as COVID or current geopolitical conflicts is presented in Supplementary Table S1, http://links.lww.com/PR9/A365. COVID-related items (T2 and T3) were partially adapted from Fallon et al.^12^ For most single items, we used a 10-cm visual analog scale (VAS) to assess individual experiences as well as self-perceived changes of pain, well-being, anxiety, and physical activity levels relative to an average prepandemic (T1–T3) (Table S1, http://links.lww.com/PR9/A365, items 17–20) or pandemic week (T2–T3) (Table S1, http://links.lww.com/PR9/A365, items 13–16). These items assessing self-perceived changes were, for example, phrased as follows: “How intense has your pain been during the past week? (Compared to T2)” (−100: Much less intense; 0: Unchanged; 100: Much more intense). The differential scores derived from this procedure were numerically transformed to a score ranging from −100, with negative integers indicative of decreases (eg, of pain), to +100, with positive integers indicating increases (0 values referred to no perceived change).

The described tools and single items fall into 4 categories: (1) sociodemographic information (ie, age, education, employment status), (2) clinical variables (ie, pain characteristics, anxiety, and depression symptoms), (3) individual experience of the pandemic and current conflicts/crises, and (4) self-perceived changes of pain, anxiety, depression, and physical activity over time. For a compilation of all used diagnostic and clinical tools as well as the associated group values obtained, see Table 1.

### 2.3. Data analysis

All data were analyzed using the SPSS software package (Version 29.0 for Windows, SPSS Inc., Chicago, IL). For our first evaluation step, we compared baseline values at T1 between participants who took part at T3 with dropouts, using 2-sample *t*-tests, to characterize the current sample. Subsequently, in all participants assessed at T3, general linear models (GLM) for repeated measures were calculated using the test values (MPI, PRSS, FSQ, CES-D) assessed at all 3 time points to map longitudinal changes. The MPI was chosen as a representative pain questionnaire and the CPG was omitted, due to the similar thematic concepts. Sphericity was tested using Mauchly test of sphericity, and Greenhouse–Geisser correction was conducted in cases where sphericity was violated. Significant effects in the GLMs were followed up using post hoc tests.

In a following step, our aim was to relate the longitudinal symptom progression to our patients' self-perception. For this purpose, self-perceived changes of pain, well-being, anxiety and physical activity levels at T3 compared with T1 and T2 were tested for statistical significance using univariate *t*-tests. Subsequently, the 2 self-perceived changes in relation with T1 (T1–T3) and T2 (T2–T3) were compared using paired *t*-tests.

To better explain symptom severity at T3 with regard to specific individual risk factors, we performed a stepwise regression analysis with a forward selection approach. Controlling for participant age and reports of other illness during the last 2 weeks, we introduced current T3 test values (MPI, PRSS, FSQ, CES-D, PCS) and single items (perceived stress and threat from current global crises and conflicts; stress from past COVID infections) as predictors and MPI pain severity at T3 as the dependent variable.

Another stepwise regression analysis with a forward selection approach and MPI pain severity at T3 as the dependent variable was calculated. To account for longitudinal trends of symptom progression over time, T1 (second step) and T2 (third step) test scores (MPI, PRSS, FSQ, CES-D, PCS [at T2]) were introduced as independent variables using a stepwise addition approach respectively.

A further stepwise regression analysis with self-perceived changes of pain severity from T2 to T3 aimed to examine the connection between increased stress levels (in this case related to fear/worries regarding current crises and conflicts) and self-perceived increases in pain.

## 3. Results

Our first evaluation step was devoted to characterizing the current sample. We therefore compared baseline values at T1 between participants who took part at T3 and those who did not (see Supplementary Table S2, http://links.lww.com/PR9/A365). Significant group differences emerged for several variables. At the time of the initial survey, T3 participants were significantly older than those who dropped out (*t* [180] = −2.98, *P* = 0.003). In addition, they reported lower pain severity (*t* [180] = −2.48, *P* = 0.014) and less pronounced pain catastrophizing tendencies (*t* [159.33] = −3.32, *P* = 0.001). At T3, around 68.8% of participants from T2 were retained. Relative to T1, retention was 36.1%, which can likely be explained by the characteristics of this specific patient group and the extended study period.

### 3.1. Characteristic experiences of patients with fibromyalgia during and after the pandemic

Part of our survey was dedicated to the individual retrospective classification of the experiences related to the COVID-19 pandemic. In this study, 78.7% of participants reported having contracted COVID at least once since our last survey period in 2022. At the same time, 52% of all patients stated that they had experienced long-term health consequences following an infection after overcoming the disease. 13.3% reported that someone in their close social circle had died from a COVID infection during the pandemic.

The following items focused on the more current experiences of our participants. 54.1% of all respondents reported suffering from another illness in addition to chronic pain during the past 2 weeks (mainly migraine, viral infections, osteoporosis, arthrosis).

80% of the patients surveyed stated that they were at least somewhat interested in current internationally relevant events, conflicts, and crises. The perceived stress caused by respective (threatening) events and conflicts was rated 61.45 ± 29.33 (out of 100) on average, with the participants also being worried that the threats could become an acute danger to them personally (M = 56.47 ± 27). An open text item confirmed that a large number of respondents were particularly concerned about the war in Ukraine.

### 3.2. Fibromyalgia display significant improvements of pain-induced interference (T2–T3) and affective distress (T1–T3)

To map longitudinal symptom trajectories, we subsequently calculated GLMs for repeated measures of all relevant test scores collected at the 3 time points. MPI pain severity scores showed no significant longitudinal change, *F* (2, 72) = 1.491; *P* = 0.232. However, descriptive assessment of MPI pain severity mean values exhibited a slight reduction in pain (T2 = 4.09, T3 = 3.84). Post hoc tests that followed significant GLMs revealed significant longitudinal reductions of pain-related interference (T1–T3; T2–T3) (*P* = 0.004; *P* = 0.007), affective distress (T1–T3) (*P* = 0.012), social activity (T1–T2) (*P* = 0.003), and general activity levels (T1–T2) (*P* = 0.01). No significant long-term changes were found for the remaining test values. All results of the above-mentioned GLMs are provided in Table 1.

### 3.3. Self-perceived longitudinal worsening of pain reported by patients with fibromyalgia

**Figure 1.**
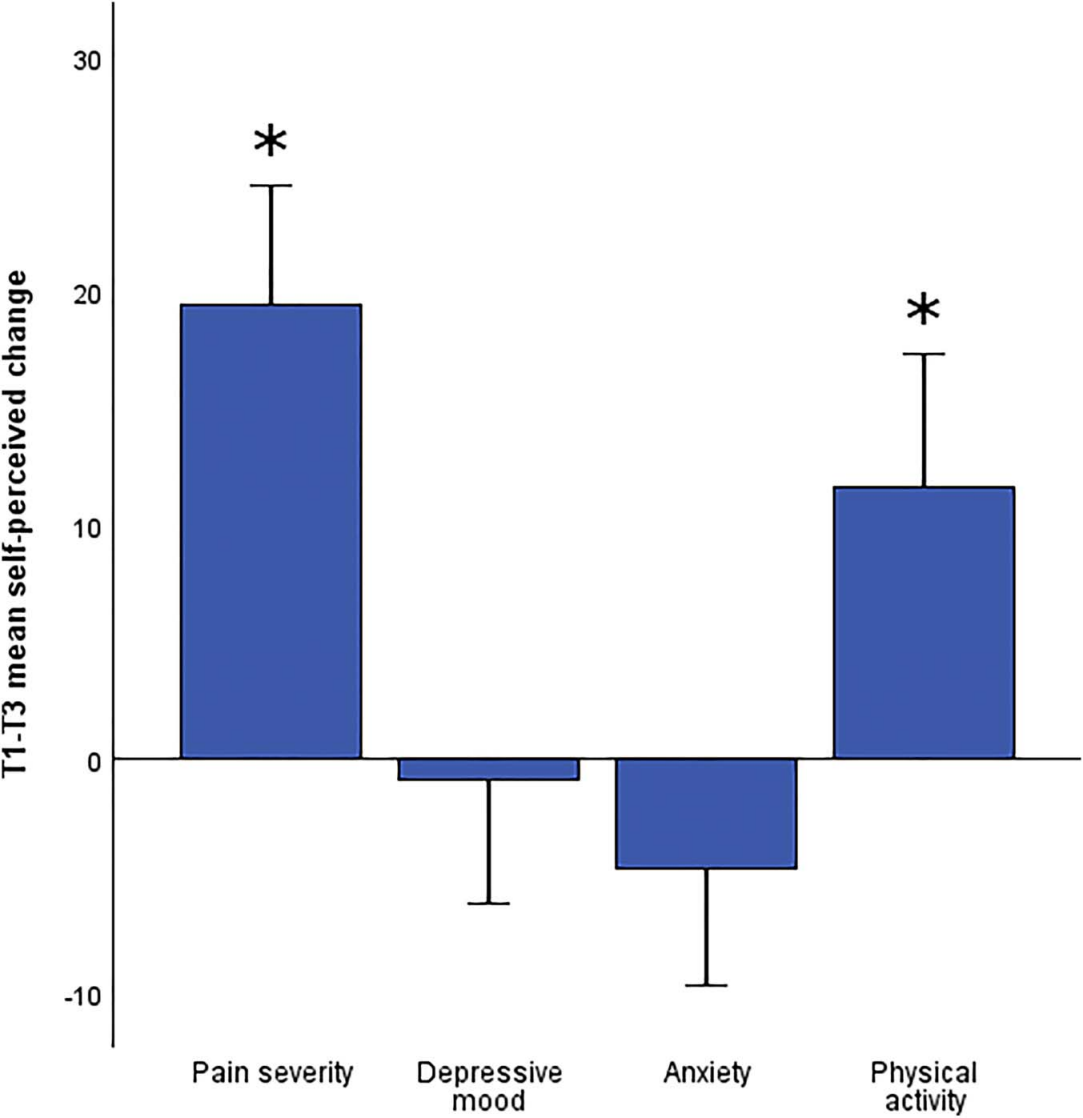
Self-perceived changes of pain intensity, depressive mood, anxiety, and physical activity compared with prepandemic levels. Perceived changes were rated on differential VAS from −100 to 100, with negative/positive integers indicating decreases/increases of the corresponding parameters. The depicted error bars illustrate the standard error of the mean. VAS, visual analog scale. *Significant change (*P* < 0.05)

We calculated univariate *t*-tests on self-perceived changes assessed at T3 (VAS from −100 to 100) of current experiences relative to an average prepandemic (T1) and pandemic week (T2). In this study, patients with FM reported statistically significant self-perceived increases in pain severity at T3 compared with T1 (mean change: 19.4 ± 43.84; *t* [74] = 3.83, *P* < 0.001) and T2 (mean change: 18.38 ± 42.09; *t* [73] = 3.76, *P* < 0.001) as well as an increase in physical activity compared with T1 (mean change: 11.6 ± 49.2; *t* [74] = 2.04, *P* = 0.045). Self-perceived changes of all other criteria in relation with T1 (T1–T3) and T2 (T2–T3) did not differ significantly (*P* between 0.475 and 0.878). For an illustration of self-perceived changes compared with prepandemic levels, see Figure 1.

### 3.4. Pain-related interference, perceived helplessness, and social activity levels at T3 predict pain severity at T3

A stepwise regression analysis used MPI pain severity at T3 as the dependent variable and several T3 test scores and single items as predictors. Participant age and reports of other illness during the last 2 weeks were included in the first model, which did not reach statistical significance (*F* [2, 51] = 0.049; *P* = 0.95), explaining 0.2% of variance. The second model introduced MPI interference at T3 as a significant predictor and explained 37.6% (corrected: 33.9%) of variance. The third model additionally included PCS helplessness at T3 as a predictor, explaining an additional 8.3% of variance (45.9%, corrected: 41.5%). Finally, the fourth model included MPI social activities at T3 as the last significant predictor and explained an additional 5% of variance (50.9%, corrected: 45.8%). This final model included MPI interference at T3, PCS helplessness at T3, and MPI social activities at T3 (MPI interference T3: β = 0.599; *P* < 0.001; PCS helplessness T3: β = 0.344; *P* = 0.004; MPI social activities T3: β = 0.266; *P* = 0.032) as significant predictors of pain severity at T3. The models described are listed in Table 2.

**Table 2:** Multiple regression model 1 of pain severity at T3 predicted by other T3 test scores and single items.

	*R*	*R* ^2^	Adj. *R*^2^	*B*	*SE*	β	*t*	*P*
Step 1	0.044	0.002	−0.037					
Age				0.002	0.02	0.016	0.11	0.913
Other illness				0.088	0.314	0.039	0.279	0.781
Step 2	0.613	0.376	0.339					
Age				0.007	0.016	0.052	0.458	0.649
Other illness				−0.063	0.253	−0.028	−0.25	0.804
MPI pain interference				0.543	0.099	0.616	5.478	**<0.001** *
Step 3	0.677	0.459	0.415					
Age				0.007	0.015	0.052	0.489	0.627
Other illness				−0.19	0.242	−0.085	−0.786	0.436
MPI pain interference				0.42	0.104	0.476	4.046	**<0.001** *
PCS helplessness				0.058	0.021	0.328	2.736	**0.009** *
Step 4	0.713	0.509	0.458					
Age				0.003	0.014	0.02	0.191	0.85
Other illness				−0.117	0.235	−0.052	−0.496	0.622
MPI pain interference				0.529	0.111	0.599	4.748	**<0.001** *
PCS helplessness				0.061	0.02	0.344	2.981	**0.004** *
MPI social activities				0.317	0.144	0.266	2.211	**0.032** *

Step 1 covers the inclusion of confound variables before the predictor analysis.

* Significant change (*P* < 0.05), marked in bold.

β, standardized; Adj. *R*^2^, corrected variance estimate; *B*, unstandardized regression coefficient; *R*^2^, variance explained by the model.

### 3.5. Pain severity levels at T1 and at T2 as well as affective distress at T2 (during COVID) predict pain severity at T3

We calculated a stepwise regression analysis with T3 pain severity as the dependent variable and several T1 and T2 test scores as independent variables. Introducing age and reports of other illness during the last 2 weeks at T3 as confound variables, the model explained only about 0.4% of variance, clearly missing statistical significance (*F* [2, 66] = 0.132; *P* = 0.88). The subsequent model included MPI pain severity at T1 as a predictor and explained 40% of variance (corrected: 37.3%). The third model added pain severity at T2 as another predictor, explaining an additional 9.4% of variance (49.4%, corrected: 46.3%). The fourth and last model included MPI affective distress at T2 (during COVID) as a predictor, explaining an additional 3.2% of variance (52.6%, corrected: 48.8%). In this final model, pain severity at T1 and T2 as well as affective distress at T2 were significant predictors (MPI pain T1: β = 0.3; *P* = 0.02; MPI pain T2: β = 0.378; *P* = 0.006; MPI affective distress: β = 0.199; *P* = 0.045) of pain severity at T3 (Table 3).

**Table 3:** Multiple regression model 2 of pain severity at T3 predicted by test scores at T1 and T2.

	*R*	*R* ^2^	Adj. *R*^2^	*B*	*SE*	β	*t*	*P*
Step 1	0.063	0.004	−0.026					
Age				0.009	0.018	0.062	0.502	0.617
Other illness				0.014	0.288	0.006	0.05	0.96
Step 2	0.633	0.4	0.373					
Age				0.004	0.014	0.028	0.29	0.773
Other illness				−0.135	0.226	−0.058	−0.598	0.552
MPI pain severity T1				0.683	0.104	0.634	6.556	**<0.001** *
Step 3	0.703	0.494	0.463					
Age				−0.003	0.014	−0.02	−0.22	0.827
Other illness				−0.211	0.21	−0.091	−1.002	0.32
MPI pain severity T1				0.344	0.138	0.319	2.493	**0.015** *
MPI pain severity T2				0.479	0.139	0.448	3.446	**0.001** *
Step 4	0.725	0.526	0.488					
Age				−0.004	0.013	−0.026	−0.29	0.773
Other illness				−0.253	0.206	−0.109	−1.224	0.226
MPI pain severity T1				0.323	0.135	0.3	2.395	**0.02** *
MPI pain severity T2				0.403	0.141	0.378	2.872	**0.006** *
MPI affective distress T2				0.168	0.082	0.199	2.044	**0.045** *

Step 1 covers the inclusion of confound variables before the predictor analysis.

* Significant change (*P* < 0.05), marked in bold.

β, standardized regression coefficient; Adj. *R*^2^, corrected variance estimate; *B*, unstandardized regression coefficient; *P*, significance value; *R*^2^, variance explained by the model; *SE*, standard error; *t*, estimated coefficient.

### 3.6. Worries that current conflicts and crises might soon pose a direct threat as well as general interest for current geopolitical events predict self-perceived changes of pain severity from T2 to T3

Another stepwise regression analysis used self-perceived changes of pain severity from T2 to T3 as the dependent variable and several single items regarding fears/worries about current conflicts and crises as independent variables. The aim of this approach was to substantiate the postulated link between increased stress levels and the observed shift in retrospective pain assessment. Introducing age and reports of other illness during the last 2 weeks at T3 as confound variables, the model explained 5% of variance and did not reach statistical significance (*F* [2, 70] = 1.89; *P* = 0.158). The second model introduced worries about a possible direct threat (Table S1, http://links.lww.com/PR9/A365, item 11) as a significant predictor and explained 11.2% (corrected: 7.3%) of variance. The third and final model additionally included general interest for current geopolitical events (Table S1, http://links.lww.com/PR9/A365, item 9) as a predictor, explaining an additional 6.4% of variance (17.6%, corrected: 12.8%) (Table 4).

**Table 4:** Multiple regression model 3 of self-perceived change of pain severity T2–T3 predicted by fears/worries about current conflicts and crises.

	*R*	*R* ^2^	Adj. *R*^2^	*B*	*SE*	β	*t*	*P*
Step 1	0.226	0.051	0.024					
Age				1.27	0.655	0.227	1.95	0.056
Other illness				−1.24	9.84	−0.015	−0.126	0.96
Step 2	0.335	0.112	0.073					
Age				0.1.05	0.646	0.187	1.62	0.109
Other illness				−4.44	9.7	−0.053	−0.457	0.649
Item 11: Worries about a possible threat				0.383	0.176	0.253	2.17	**0.033** *
Step 3	0.42	0.176	0.128					
Age				1.63	0.676	0.29	2.41	**0.019** *
Other illness				−6.7	9.46	−0.079	−0.708	0.482
Item 11: Worries about possible threat				0.602	0.196	0.397	3.08	**0.003** *
Item 9: General interest for current events				−0.486	0.211	−0.316	−2.3	**0.024** *

Step 1 covers the inclusion of confound variables before the predictor analysis.

* Significant change (*P* < 0.05), marked in bold.

β, standardized regression coefficient; Adj. *R*^2^, corrected variance estimate; *B*, unstandardized regression coefficient; *P*, significance value; *R*^2^, variance explained by the model; *SE*, standard error; *t*, estimated coefficient.

## 4. Discussion

Various studies have demonstrated a wide range of extensive negative consequences of the COVID-19 pandemic on the physical and mental well-being of people around the world. This particularly applies to vulnerable groups, such as patients with chronic pain. A key finding from our previous study was that patients experienced a broad self-perceived worsening of pain symptoms and well-being related to the pandemic, while longitudinal reference values from pain questionnaires showed no significant change. Based on this observation, we argued that self-perceived increases of pain may reflect a behavioral correlate of psychological distress rather than a measurable worsening of physical pain. One of this study's main objectives was to further investigate the relationship between self-perceived and actual longitudinal changes in chronic pain symptoms throughout the postpandemic period.

### 4.1. Characteristic burdens reported by patients with fibromyalgia and the aftermath of the pandemic

With regard to the pandemic period, the majority of patients reported that they had experienced long-term health consequences following a COVID-19 infection. This seems plausible, as patients with FM represent a vulnerable group, frequently affected by severe courses of disease and long-term complications. In this context, the frequently discussed similarities and parallels between FM and long-COVID are worth mentioning.^8,13^ It has even been speculated that SARS-CoV-2 might contribute to the development of FM or exacerbate its symptoms as both syndromes share similar mechanisms and clinical manifestations.^13^ The longitudinal course of these symptoms within our sample will be discussed in the following sections.

Participants reported high levels of perceived distress related to internationally relevant events, conflicts, and crises and were largely concerned that conflicts may become a threat to them personally. Based on these findings, it can be assumed that wars and geopolitical crises (among other topics) might have replaced the pandemic as a global societal stressor, as some studies already suggest.^5,31^

### 4.2. Longitudinal changes of pain and other clinical measures in patients with fibromyalgia

As with our preceding investigation and the comparison of T1 with T2, patients with FM experienced no measurable longitudinal worsening of pain severity from T2 to T3. On the contrary, significant improvements regarding pain-related interference and affective distress for the postpandemic period leading up to our recent survey were observed. According to this, certain degrees of symptom relief were observed following the pandemic. This could be due, at least in part, to the return to normal social conditions, as social connection is generally considered to be of vital importance in shaping the painful experience.^19,36^

### 4.3. Self-perceived vs longitudinal T1 to T3 changes of pain severity

Similar to our preceding investigation, patients with FM experienced significant self-perceived worsening of pain severity, both in comparison with the last examination period in 2022 as well as to the initial prepandemic survey phase, which was not reflected in our longitudinal data. These new findings suggest that the self-perceived worsening of pain symptoms, demonstrated in our preceding study^25^ and numerous other studies,^7,19,27,32^ was not necessarily a consequence of the pandemic and the associated lockdown periods. Rather, self-perceived aggravation of pain presumably reflects more generalized retrospective misjudgment that appears to be characteristic for our patient group—as shown for all possible retrospective comparisons (T1–T2, T1–T3 and T2–T3). Such retrospective assessment can be influenced by a variety of factors, such as the experience of interference, psychological distress, and catastrophizing tendencies. It should be noted here that not all self-perceived retrospective measures deviate from longitudinal changes. Accordingly, we assume that the differences do not occur due to systematic survey errors but rather, as mentioned above, to a cognitive bias. Evidence of recall bias in retrospective pain assessments of chronic pain patients has already been found earlier.^16^ This assumption is further supported by our finding that self-perceived changes in pain intensity were predicted by fears/concerns surrounding current conflicts and crises.

As patients in our previous study perceived a pandemic-related worsening of pain, while MPI pain severity scores did not change from T1 to T2, we originally argued that self-perceived increases of pain might reflect a behavioral correlate of psychological distress rather than a measurable longitudinal change of physical pain. Our new findings suggest that such behavioral correlates occur more frequently than expected and are not exclusively linked to psychological distress caused by the pandemic. This is supported by the observation that, even at T3, participants continuously reported high levels of stress, which they attributed to ongoing conflicts and crises. These results indicate that psychological distress, regardless of its origin, may play a key role in modulating pain perception in patients with FM. It should be noted in this context that the pandemic is unlikely to constitute a societal stressor of unique magnitude. Rather, in the ensuing years, the pandemic was followed by geopolitical crises and conflicts that exert a similarly stress-inducing impact on individuals.

### 4.4. Predictors of pain severity at T3

A stepwise regression analysis using T3 test scores and single items as independent variables and T3 pain severity as the dependent variable yielded 3 significant predictors: pain-related interference, the feeling of helplessness, and social activity levels. For all of these factors, previous studies have shown an influence on the painful experience in patients with chronic pain.^3,17,21,26,30^ With regard to social activity levels, it should be mentioned that most previous studies examined related categories such as “social support.” In comparison, our category “social activities” (MPI) represents a more active lifestyle aspect. In view of the COVID-19 pandemic and its associated restrictions on social life, the influence of social activity levels on pain severity is of particular interest. The findings could thus be interpreted as further evidence of widespread adverse effects of the pandemic, particularly for vulnerable groups such as chronic pain patients. However, it is important to note that no unilateral causal relationship can be assumed for the reported factors. For example, chronic pain has previously been shown to lead to social withdrawal and thus a possible reduction in social activity levels.^10,20^

A subsequent stepwise regression analysis used T1 and T2 data as predictors for T3 pain. In this analysis, pain severity at T1 and T2 as well as affective distress at T2 (during COVID) were significant predictors of current pain. This initially confirms some of the observations from our preceding investigation,^25^ in which T1 pain severity was the strongest predictor of pain at T2. Therefore, this effect remains unchanged with continuous observation and longer observation periods. Finally, affective distress experienced during the pandemic (at T2) had a decisive impact on pain severity at T3, whereas affective distress at T3 had no significant predictive influence. This finding is of particular interest to us, as it suggests that affective well-being during the pandemic, or the lack thereof, is still reflected in the current manifestation of pain symptoms, providing evidence for prolonged adverse effects related to affective distress during the pandemic. These findings seem particularly relevant, as numerous studies on patients with FM have shown a sharp increase in affective distress during the pandemic.^23,28^ In this context, it seems especially significant to intervene therapeutically at the earliest possible stage. Cognitive behavioral therapy has repeatedly been proven to provide great support regarding affective distress and pain catastrophizing.^30^

In light of these findings, it should be noted that the T3 sample represents the less pain-affected portion of the original T1 population. Therefore, it cannot be determined with certainty to what extent the reported results reflect the symptom trajectories of patients who were more severely affected at baseline.

## 5. Conclusion

Although the observed changes are not necessarily attributable solely to the COVID-19 pandemic and related factors, our comprehensive survey allows to draw cautious conclusions regarding long-term symptom development in FM during and beyond the pandemic. As with our previous investigation at T2, patients displayed no measurable increase of pain severity compared with the last examination, while reporting a significant self-perceived worsening of pain. This underpins the findings from our preceding study, underlining the importance of targeting psychological factors, specific needs, and issues that might modulate the painful experience. Our current data also emphasize the modulatory influence of social contacts/activity on the development of pain symptoms. Accordingly, maintaining a healthy social environment and activity level should implicitly be integrated into therapy concepts as a salutogenic factor. Current pain was also determined by the amount of affective distress experienced during the pandemic. This illustrates the far-reaching and long-lasting adverse consequences observed due to high levels of psychological burden.

## Disclosures

The authors do not have any conflicts of interest, either financial or otherwise related directly or indirectly to this article.

## Supplemental digital content

Supplemental digital content associated with this article can be found online at http://links.lww.com/PR9/A365.

## Supplementary Material
