# Supplementary material for: Long-term examination of pain and health-related outcomes in people with fibromyalgia before, during, and after COVID-19
Source: Pain Rep. 2025 Dec 18;11(1):e1380. doi: 10.1097/PR9.0000000000001380 (PMC12721768; doi:10.1097/PR9.0000000000001380)
Supplement: Supplementary file 1 [file painreports-11-e1380-s001.pdf]

Supplementary material

Table S1. Single items introduced in this study.

1. Have you contracted COVID-19 at least once in the past 2 years (since 2022)?<br>If yes: How many times?<br>If yes: How stressful did you perceive the illness to be?
2. Did you experience any adverse long-term health consequences after recovering from the illness?<br>If yes: For how long?
3. Has anyone in your close social circle died due to a COVID-19 infection in the last two years?<br>If yes: What was your relationship to this person (family, friends, acquaintances, etc.)?
4. In the last two weeks, have you suffered from any other medical condition in addition to your pain?<br>If yes: What illness?
5. How intense has your pain been in the past week (on average)?
6. How exhausted have you felt in the past week?
7. How lonely have you felt in the past week?
8. How anxious have you felt in the past week?
9. How interested are you in current events in Germany and the world?<br>(Conflicts and crises such as the Ukraine war, the Middle East conflict, climate change, infectious diseases, the political situation in Germany, etc.)
10. How stressful do you perceive the current conflicts and crises in the world to be?
11. Are you worried that the current conflicts and crises in the world could become a threat to you personally?
12. Which topic has particularly occupied your thoughts throughout the past two years?<br>How stressful did you perceive this topic overall?
13. How tense, nervous, or anxious have you felt in the past week? (Compared to T2)
14. How depressed/down have you felt in the past week? (Compared to T2)
15. How much physical activity did you engage in during the past week? (Compared to T2)
16. How intense has your pain been during the past week? (Compared to T2)
17. How tense, nervous, or anxious have you felt in the past week? (Compared to T1)
18. How depressed/ down have you felt in the past week? (Compared to T1)
19. How much physical activity have you done in the past week? (Compared to T1)
20. How intense has your pain been during the past week? (Compared to T1)

Exemplary VAS rating from -100 to 100 (Item 16):

16. How intense has your pain been during the past week? (Compared to T2)

Much less intense Unchanged Much more intense

Table S2. T1 comparisons of participants who took part at T3 and those who dropped out.

	T3 sample		Drop-outs		*t*-test	
	M	*SD*	*M*	*SD*	*t*-value (df)	*p*
Age (years)	53.01	7.67	49.03	9.66	2.98 (180)	**.003***
CES-D	22.82	7.25	24.71	6.95	-1.75 (152.76)	.082
FSQ						
Symptom Severity Score	9.46	2.14	9.86	1.7	-1.33 (135.9)	.185
Widespread Pain Index	11.33	4.62	11.39	3.91	-.087 (142.67)	.931
PRSS						
Catastrophizing	2.27	1.1	2.82	1.1	-3.32 (159.33)	**.001***
Coping	3.02	0.91	3.05	0.76	-.228 (140.63)	.82
MPI						
Pain severity	3.93	1.11	4.29	0.84	-2.48 (180)	**.014***
Interference	4.16	1.22	4.5	1	-1.96 (138.6)	.052
Life control	3.21	1.36	2.99	1.22	1.12 (148.41)	.263
Affective distress	3.61	1.36	3.53	1.33	.394 (156.99)	.694
Social support	3.27	1.69	3.5	1.7	-.89 (157.62)	.376
Punishing responses	1.32	1.6	1.62	1.59	-1.23 (154.82)	.221
Solicitous responses	3.03	1.67	3.34	1.53	-1.22 (147.15)	.225
Distracting responses	2.6	1.45	3	1.36	-1.83 (149.46)	.069
Social activities	2.38	0.94	2.22	1.02	1.1 (167.14)	.273
General activity level	7.61	2.4	7.28	2.54	.874 (164.94)	.383

Note. *M*: mean; *SD*: standard deviation; df: degrees of freedom; CES-D: Center for Epidemiologic Studies Depression Scale; FSQ: Fibromyalgia Survey Questionnaire; PRSS: Pain-related Self Statements Scale; MPI: West Haven-Yale Multidimensional Pain Inventory. * significant change ($p < .05$).
